# In Vitro Study of Combined Application of Bevacizumab and 5-Fluorouracil or Bevacizumab and Mitomycin C to Inhibit Scar Formation in Glaucoma Filtration Surgery

**DOI:** 10.1155/2019/7419571

**Published:** 2019-10-27

**Authors:** Yuanyuan Zhang, Shaopin Zhu, Xun Xu, Lei Zuo

**Affiliations:** ^1^Department of Ophthalmology, Shanghai Fourth People's Hospital, Tongji University School of Medicine, Shanghai 200081, China; ^2^Department of Ophthalmology, Shanghai General Hospital, Shanghai Jiao Tong University School of Medicine, Shanghai 200080, China

## Abstract

This is an in vitro study conducted to observe the safety and antiscarring effects of combined application of bevacizumab (BVZ) and 5-fluorouracil (5-Fu) or BVZ and mitomycin C (MMC) during glaucoma filtration surgery (GFS). The cytotoxicity of drug combinations in human Tenon's fibroblasts (HTFs) and human umbilical vein endothelial cells (HUVECs) was evaluated. Their effects on the levels of vascular endothelial growth factor (VEGF) in HUVECs, cell proliferation and migration in HTFs, and the expression of collagen type I alpha 1 (Col1A1) gene in HTFs were evaluated. In addition, the effects of combined drugs on VEGF(R) mRNA in HTFs were detected to explore the possible underlying drug mechanisms. The results showed that BVZ combined with 5-Fu demonstrated more significant antiscarring effects than BVZ or 5-Fu alone. However, the inhibitory effects of BVZ combined with MMC were similar to those of MMC alone. The cytotoxicity of the drug combinations was significantly greater than that of single drug, suggesting that combined application of BVZ and antimetabolites after GFS was safer when applied at different sites (such as subconjunctival injection at bilateral sides of the filtering bleb) or at varied time points.

## 1. Introduction

The key to a successful outcome of glaucoma filtration surgery (GFS) is by achieving wound healing inhibition [1]. To reduce scar formation after trabeculectomy, maintain continuous filtration, and reduce intraocular pressure, antimetabolites such as 5-fluorouracil (5-Fu) and mitomycin C (MMC) are applied during surgery, which improves the success rate of the surgery to a certain extent [2]. In 2006, utilization of needle bleb revision with bevacizumab in a patient with a failing bleb following trabeculectomy is explored [3]. A subconjunctival injection of a certain dose of bevacizumab (BVZ) is given for inhibiting scar formation during trabeculectomy [4].

The concentration of vascular endothelial growth factor (VEGF) is increased for all ocular diseases such as neovascularization and/or inflammation, such as proliferative diabetic retinopathy [5], neovascular glaucoma [6], uveitis [7], and age-related macular degeneration [8]. In addition, VEGF is also associated with fibrosis and inflammation [9, 10] and plays an important role in scar formation after GFS [11].

VEGF induces proliferation of Tenon's fibroblasts in vitro during posttrabeculectomy wound healing process. Bevacizumab reduces the proliferation of fibroblasts in vitro and improves the surgical outcome [12]. Moreover, the water-tight suturing of the conjunctiva counteracts with BVZ-induced delayed healing of conjunctival wounds [13]. Subconjunctival injection of BVZ reaches an effective level in the intraocular tissues of the treated eyes [14]. Based on these studies, subconjunctival injection of a certain dose of BVZ for inhibiting scar formation during trabeculectomy has been used [4, 15, 16].

As the scar formation after GFS involves complex processes of angiogenesis and fibrosis, it is inadequate to aim only on anti-VEGF or other single targets (such as antitransforming growth factor beta-2 and TGF-*β*2) [17]. However, the use of antimetabolites is associated with several sight-threatening complications [18, 19]. Therefore, clinicians have combined the application of BVZ and MMC or BVZ and 5-Fu to improve the clinical outcomes of GFS for refractory glaucoma [20, 21], trying to reduce the risk of antimetabolite use and effectively inhibit scar formation after GFS. Spitzer et al. [22] have reported on the toxicity of BVZ on human ocular cells; the safety of BVZ combined with 5-Fu (BVZ + 5-Fu) and BVZ combined with MMC (BVZ + MMC) in human ocular cells still remains unclear. The effects of BVZ combined with 5-Fu or MMC on VEGF levels in vascular endothelial cells on cell proliferation and migration and on collagen formation in human Tenon's fibroblasts (HTF) [23] are still rarely reported.

Hence, this study aimed to evaluate the safety and antiscarring effects of BVZ + 5-Fu and BVZ + MMC in GFS through in vitro experiments. Also their effects on cytotoxicity, cell proliferation and migration, and collagen formation (collagen type I alpha l, Col1A1) [24] were observed, and their effects on VEGF and VEGF(R) mRNA were tested to explore the mechanisms of drug combinations. Our study assists in more comprehensively understanding the role of BVZ combined with antimetabolites during the wound healing process.

## 2. Materials and Methods

### 2.1. Cell Culture, Main Drugs, and Reagents

According to the Declaration of Helsinki, HTF cells were obtained from the specimens by excising the Tenon's capsule during strabismus surgery [24]. This study was approved by the Ethics Committee of Shanghai Fourth People's Hospital affiliated to Tongji University School of Medicine (Approval No. 2019012). Rabbit anti-human vimentin monoclonal antibodies (1 : 250, AB92547, Abcam) and rabbit anti-human keratin monoclonal antibodies (1 : 50, EPR17882, Abcam) were purchased from Abcam (Cambridge, England). HTF was cultured in Dulbecco's modified Eagle's medium (DMEM) supplemented with 10% fetal bovine serum and antibiotics. Human umbilical vein endothelial cells (HUVECs) (CRL-2873; American Type Culture Collection [ATCC]) were cultured in DMEM/F12 medium containing 10% fetal bovine serum and antibiotics. Culture media, antibiotics, trypsin (1 : 250), recombinant human vascular endothelial growth factor (VEGF), 4′,6-diamidino-2-phenylindole (DAPI), 3-(4,5-dimethylthiazol-2-yl)-2,5-diphenyltetrazolium bromide (MTT), and heat-inactivated fetal bovine serum (FBS) were obtained from Invitrogen (Carlsbad, CA). Endothelial cell growth medium was purchased from PromoCell GmbH (Heidelberg, Germany). Bevacizumab (Avastin), phosSTOP, and protease inhibitors were obtained from Roche (Basel, Switzerland). The ELISA kit was brought from R&D Systems (Minneapolis, Minnesota, USA). A Bradford protein assay kit was brought from Bio-Rad (Hercules, California, USA). Fluorouracil (25 mg/ml) was purchased from Shanghai XudongHaipu Pharmaceutical Co., Ltd., and mitomycin was purchased from Zhejiang Haizheng Pharmaceutical Co., Ltd. Phosphate buffer solution (PBS) and 0.9% sodium chloride were obtained from Baxter Medical Products Co., Ltd.

### 2.2. MTT Assay for Cytotoxicity/Proliferation of HUVECs and HTF

Single cell suspension cultured under normal conditions at logarithmic growth phase was inoculated into 6-well culture plates at a density of 5 × 10^4^ per well and synchronized with serum-free RPMI 1640 medium. HUVECs were incubated with BVZ, 5-Fu, MMC, BVZ/5-Fu, BVZ/MMC, or PBS (control) for 24 h, and HTF cells were incubated with BVZ, 5-Fu, MMC, BVZ/5-Fu, BVZ/MMC, or PBS (control) for 24 h (the concentrations of medications are shown in Table 1). The cells were rinsed with PBS and then fresh serum-free medium with or without 0.5 mg/mL MTT was added into the cells. After 2 h of incubation, an amount of formazan was extracted and colorimetric assay of ELISA (Emax, Molecular Devices Corporation, Sunnyvale, California, USA) was performed to determine the absorbance value per well at 570 nm [25].

### 2.3. Determination of VEGF Levels in HUVECs

HUVECs were treated with 5-Fu (0.5 mg/ml), MMC (0.0002 mg/ml), BVZ (0.25 mg/ml), 5-Fu (0.5 mg/ml) + BVZ (0.25 mg/ml), MMC (0.0002 mg/ml) +BVZ (0.25 mg/ml), or PBS. After 24 h, 200 *μ*l of supernatant was collected from each well and analyzed by a VEGF ELISA kit (R&D Systems, USA) according to the manufacturer's instructions [26].

### 2.4. Analysis of HTF Cell Migration

When the HTFs reached 80% confluence in vitro, then scratches were drawn vertical to that of a pre-drawn line with a 1 mm tip at the bottom of the culture dish, and 3 scratches were drawn with the same distance. The cells that were floating along the scratches were washed with PBS, photographed under a microscope (microscope: Leica DM IRB, magnification ratio ×40), and the time point was recorded as 0 h. A total of 6 images at different fields of view were taken. This was followed by the addition of 0.2% FBS medium and treatment of cells with 5-Fu (0.5 mg/ml), MMC (0.0002 mg/ml), BVZ (0.25 mg/ml), 5-Fu (0.5 mg/ml) + BVZ (0.25 mg/ml), or MMC (0.0002 mg/ml) + BVZ (0.25 mg/ml). Then, 30 ng/ml VEGF was added into the treatments, while 30 ng/mL of VEGF alone was added into 0.2% FBS culture medium, which acts as a positive control. The culture medium without drug intervention was set as the blank control group. The cells were further cultured and images were taken after culturing for 24 h. The same region was selected for each repetition when taking the photographs. Image J software was used for analyzing the scratch area. The wound closure rate was calculated using the formula: wound closure rate = (area of the wound at 0 h—area of the wound at 24 h)/area of the wound at 0 h [27].

### 2.5. Quantitative Polymerase Chain Reaction (PCR) Analysis of Collagen (Col1A1) and VEGF(R) mRNA in HTF

HTF cells were cultured in vitro, and the expression of VEGF, VEGFR1 (Flt-1), VEGFR2 (KDR), and collagen (Col1A1) mRNA was quantified using PCR (*n* = 3). The cells were treated with 5-Fu (0.5 mg/ml), MMC (0.0002 mg/ml), BVZ (0.25 mg/ml), 5-Fu (0.5 mg/ml) + BVZ (0.25 mg/ml), MMC (0.0002 mg/ml) + BVZ (0.25 mg/ml), or PBS. The total mRNA was extracted by using TRIzol reagent (Invitrogen, Carlsbad, CA). cDNA was then synthesized by reverse transcription (Tetro cDNA Synthesis Kit, Bioline, London, UK), and mRNA was detected by RT-PCR (SensiFAST^TM^ SYBR® Hi-ROX Kit, Bioline, London, UK) by using a special software (ABI Prism 7500 SDS Software, USA). The primers and probes for RT-PCR (Table 2) were designed by Shanghai Generay Biotech CO., Ltd. The expression levels of VEGF, Flt-1, KDR, and Col1A1 mRNA were normalized to glyceraldehyde-3-phosphate dehydrogenase (GAPDH) mRNA.

### 2.6. Statistical Analysis

The variables were described as mean ± standard deviation. If the variance was homogeneous, then least significant difference (LSD) and Student–Newman–Keuls (SNK) tests were used for analysis of variance. If the differences were homogeneous, the differences between the experimental groups were analyzed by using the rank sum test. All statistical analyses were performed by using SPSS 19.0 (SPSS Inc., IL, USA). *P* < 0.05 was considered to be statistically significant.

## 3. Results

### 3.1. Identification of HTF

**Figure 1:**
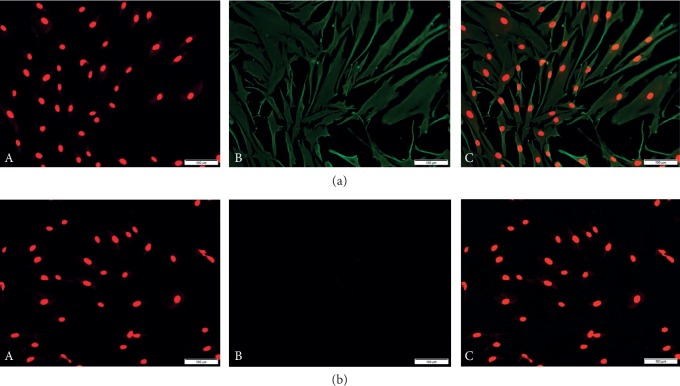
Identification of fibroblasts by immunofluorescence assay (magnification ratio ×200). (a). The nucleus of cells stained with DAPI showed red fluorescence (A), the cytoplasm of cells stained with anti-vimentin antibody showed green fluorescence (B), and combined staining images of nucleus and cytoplasm (C). (b). The nucleus stained by DAPI showed red fluorescence (A), while the cytoplasm stained with anti-keratin antibody was negative (B), and the combination of nucleus and cytoplasm staining images (C).

Immunofluorescence assay was performed to identify HTF cells. The result of staining of anti-vimentin antibody was positive, anti-keratin antibody was negative, and morphological observation of these cells confirmed them as fibroblasts [28] (see Figure 1).

### 3.2. Cytotoxicity in HTFs and HUVECs

After BVZ 0.025 mg/ml was added to the cultured HTFs, no significant cytotoxicity was observed when compared with that in the PBS group. With increasing concentration of BVZ from 0.25 to 2.5 mg/ml, cytotoxicity showed a significant increase. In the groups of MMC 0.0002, 0.002, and 0.02 mg/ml with BVZ 0.25 mg/ml, the survival ability of HTF cells was significantly lower than that in the MMC intervention group. After adding BVZ 0.25 mg/ml to 5-Fu 0.05, 0.5, and 5 mg/ml, the number of dead HTFs was also relatively increased.

**Figure 2:**
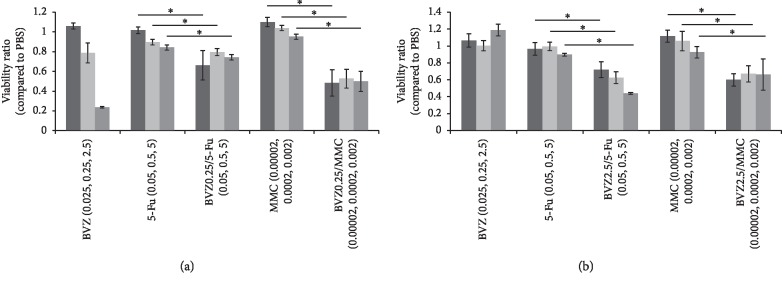
Cell viability of human Tenon's fibroblasts (HTFs) and human umbilical vein endothelial cells (HUVECs) after treatment with bevacizumab (BVZ), mitomycin C (MMC), 5-fluorouracil (5-Fu), BVZ/MMC, and BVZ/5-Fu. The cell viability of the control group was set to 100%. Unit: mg/ml. ^*∗*^*P* < 0.05. (a) Toxicity assay of HTF (24 h). (b) Toxicity assay of HUVEC (24 h).

For HUVECs, after the addition of 0.025, 0.25, and 2.5 mg/mL BVZ, the viability of HUVECs was not significantly lower than that in the PBS group. After addition of BVZ 2.5 mg/ml to MMC 0.0002, 0.002, and 0.02 mg/ml group and addition of BVZ 2.5 mg/ml to 5-Fu 0.05, 0.5, and 5 mg/ml group, the number of dead HUVECs was significantly increased than that in the 5-Fu or MMC groups without BVZ (see Figure 2 and Tables 3 and 4).

### 3.3. VEGF Levels in HUVECs

**Figure 3:**
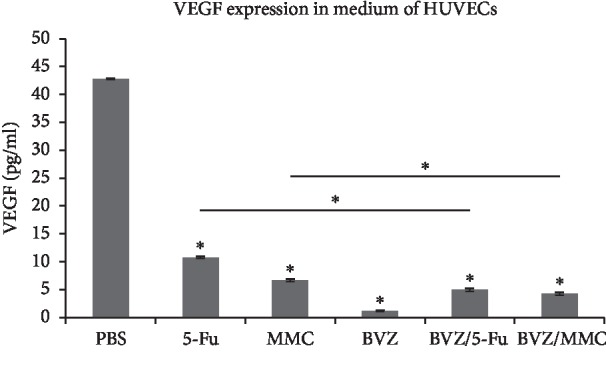
Effect of 5-fluorouracil (5-Fu), mitomycin C (MMC), bevacizumab (BVZ), BVZ/5-Fu, and BVZ/MMC on vascular endothelial growth factor (VEGF) levels in human umbilical vein endothelial cells (HUVECs) ^*∗*^*P* < 0.05.

HUVECs were cultured for 24 hours, and the VEGF levels in the control group and 5-Fu (0.5 mg/ml), MMC (0.0002 mg/ml), BVZ (0.25 mg/ml), 5-Fu (0.5 mg/ml) + BVZ (0.25 mg/ml), and MMC (0.0002 mg/ml) + BVZ (0.25 mg/ml) groups were 42.8 ± 0.12, 10.8 ± 0.21, 6.67 ± 0.23, 1.18 ± 0.11, 4.95 ± 0.26, and 4.27 ± 0.28 pg/ml, respectively. The levels of VEGF in HUVECs of BVZ, 5-Fu, and MMC intervention groups were significantly lower than those of the control group. However, the levels of VEGF in 5-Fu or MMC group with the addition of BVZ were significantly lower than those in the 5-Fu or MMC group without BVZ (Figure 3).

### 3.4. Cell Migration in HTFs

**Figure 4:**
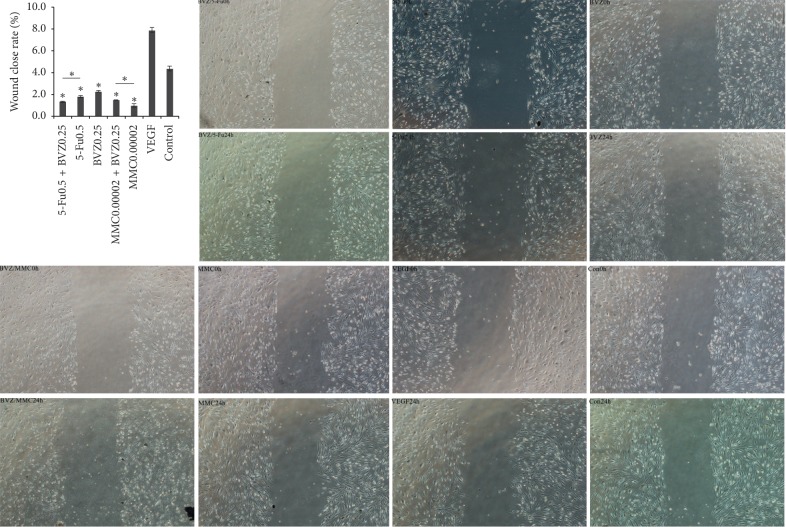
Cell migration analysis. The scratch-wound assays showed the effect of 5-fluorouracil (5-Fu), mitomycin C (MMC), bevacizumab (BVZ), BVZ + 5-Fu, and BVZ + MMC on cell migration of human Tenon's fibroblasts (HTFs) under the action of vascular endothelial growth factor (VEGF) (magnification ratio ×40). ^*∗*^*P* < 0.05.

The relative rate of HTF cell migration at 24 hours in 5-Fu (0.5 mg/ml) + BVZ (0.25 mg/ml), 5-Fu (0.5 mg/ml), BVZ (0.25 mg/ml), MMC (0.0002 mg/ml) + BVZ (0.25 mg/ml), MMC (0.0002 mg/ml) (both simultaneously adding VEGF 30 ng/ml or only VEGF 30 ng/ml) groups, and the blank control group was 0.0132 ± 0.0005, 0.0182 ± 0.0009, 0.0227 ± 0.0006, 0.0148 ± 0.0001, 0.0101 ± 0.0014, 0.0790 ± 0.0023, and 0.0439 ± 0.0018, respectively. Both drug intervention and combined drug intervention showed significant inhibitory effects on HTF cell migration. The relative rate of cell migration in the BVZ + 5-Fu group showed no significant differences from that of the MMC or BVZ + MMC group. However, the relative rate of cell migration in the MMC group was significantly lower than the BVZ + MMC group. The relative rate of cell migration in the BVZ + 5-Fu group was significantly lower than that of the BVZ or 5-Fu groups (see Figure 4 and Table 5).

### 3.5. Col1A1 mRNA Expression in HTF

**Figure 5:**
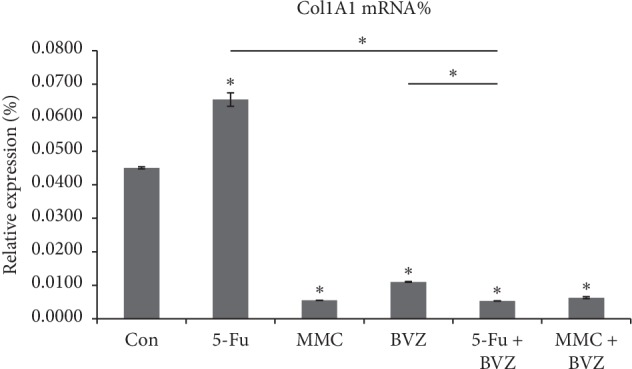
Effect of 5-fluorouracil (5-Fu), mitomycin C (MMC), bevacizumab (BVZ), BVZ + 5-Fu, and BVZ + MMC on type I collagen alpha 1 (Col1A1) mRNA in human Tenon's fibroblasts (HTFs). ^*∗*^*P* < 0.05.

The levels of Col1A1 mRNA expression in HTFs that were cultured for 24 hours in the control group and 5-Fu, MMC, BVZ, BVZ + 5-Fu, and BVZ + MMC groups were 0.0450 ± 0.0003, 0.0654 ± 0.0020, 0.0055 ± 0.0000, 0.0110 ± 0.0001, 0.0053 ± 0.0001, and 0.0063 ± 0.0003, respectively. Except for 5-Fu group, both drug intervention and combined drug interventions significantly inhibited Col1A1 mRNA expression in HTF cells. The suppression of collagen formation by BVZ + 5-Fu was significantly greater than 5-Fu or BVZ, while the inhibition of collagen production by BVZ + MMC showed no significant differences from that of MMC or BVZ + 5-Fu (see Figure 5).

### 3.6. VEGF(R) mRNA Expression in HTFs

**Figure 6:**
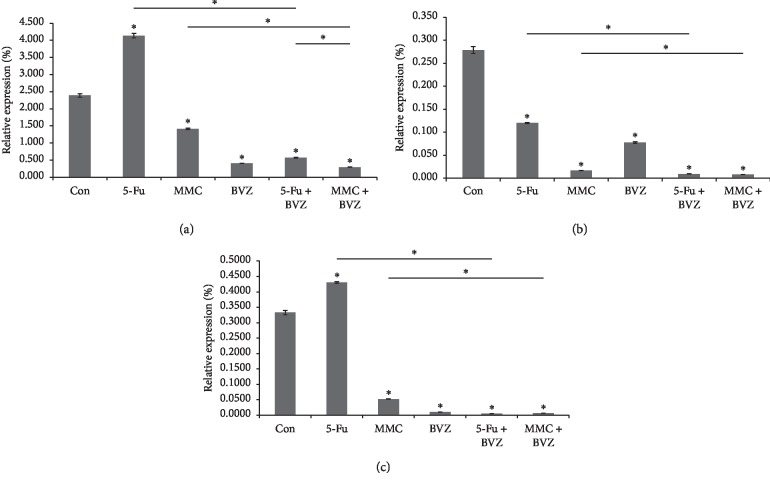
Effect of 5-fluorouracil (5-Fu), mitomycin C (MMC), bevacizumab (BVZ), BVZ + 5-Fu, and BVZ + MMC on vascular endothelial growth factor (VEGF), VEGFR1 (Flt-1), and VEGFR2 (KDR) mRNA in human Tenon's fibroblasts (HTFs). ^*∗*^*P* < 0.05. (a) Flt-1 mRNA%. (b) KDR mRNA%. (c) VEGF mRNA%.

VEGFR1(Flt-1) mRNA expression levels of HTFs cultured for 24 hours in the blank control group and 5-Fu, MMC, BVZ, BVZ + 5-Fu, and BVZ + MMC groups were 2.388 ± 0.0500, 4.137 ± 0.0678, 1.412 ± 0.0211, 0.408 ± 0.0040, 0.569 ± 0.0139, and 0.294 ± 0.0039, respectively; VEGFR2(KDR) mRNA expression levels were 0.279 ± 0.0074, 0.120 ± 0.0010, 0.017 ± 0.0003, 0.078 ± 0.0020, 0.009 ± 0.0002, and 0.008 ± 0.0001, respectively; and VEGF mRNA expression levels were 0.3329 ± 0.0072, 0.4306 ± 0.0032, 0.0521 ± 0.0005, 0.0095 ± 0.0003, 0.0050 ± 0.0001, and 0.0064 ± 0.0003, respectively. Except for the 5-Fu group, both drug intervention and combined drug intervention significantly downregulated VEGF(R) mRNA expression in HTFs. 5-Fu significantly inhibited VEGFR2 mRNA expression in HTFs only. The inhibitory effects of BVZ + 5-Fu or BVZ + MMC combined drugs were greater than those of 5-Fu or MMC single drug. However, the inhibitory effects of BVZ + 5-Fu showed no significant differences when compared with those of BVZ + MMC on VEGF mRNA and VEGFR2 mRNA expression in HTFs (see Figure 6).

## 4. Discussion

This in vitro study was conducted to investigate the safety of BVZ + 5-Fu and BVZ + MMC and their inhibitory effects on scar formation after GFS. The cytotoxicity of BVZ + 5-Fu and BVZ + MMC on HTFs and HUVECs was examined. The results reveal that BVZ + 5-Fu and BVZ + MMC can significantly inhibit the VEGF levels in HUVECs, prolong the proliferation and migration of HTFs, and inhibit the formation of collagen (Col1A1) in HTFs. Also, the effect of combined drugs on VEGF(R) mRNA in HTF cells was observed.

The surgical goal of GFS is to create an incision to bypass the trabecular meshwork and drain the aqueous humor outwards through subconjunctival filtering blebs, thereby relieving the elevated intraocular pressure [29]. Increased angiogenesis in the conjunctiva and migration of fibroblasts lead to fibroblast proliferation and concomitant collagen deposition, directly causing filtering bleb failure [30]. Angiogenesis is defined as a process of formation of new blood vessels from the existing blood vessels. It is an important process that occurs naturally during growth, reproduction, and wound healing in order to supply nutrients and oxygen to the tissues [31]. VEGF is considered to be the most common stimulator of endothelial growth and vascular permeability and might be the link between angiogenesis and scar formation [11]. VEGF not only regulates fibrosis via angiogenesis, but also acts as a mediator in signaling pathways, promoting fibroblast migration, proliferation, and collagen production [31, 32]. The wound healing response can be divided into two different stages in a mouse GFS model [33]. The early “acute inflammation” phase, which is characterized by a marked increase in the transcriptional expression of VEGF, chemokine (C-X-C motif) ligand (CXCL), and matrix metalloproteinase (MMP), as well as increased inflammatory cell infiltration. The “late fibrosis” phase is characterized by significant elevation of the expression of transforming growth factor (TGF)-*β*2 and extracellular matrix genes, with a concurrent reduction in the inflammatory cell infiltration. VEGF-A is the only isoform of VEGF that significantly decreases the expression during the late stage of wound healing process [33], suggesting that it might be involved in the transition from the early to late phases of wound healing processes [11]. VEGF signaling is involved in angiogenesis and fibrosis, which are the two key processes during GFS scar formation [11, 33].

Based on these studies, anti-VEGF therapies including BVZ are expected to delay the healing process of filtering blebs after GFS. Anti-VEGF adjuncts are preliminarily tested in vitro [34], animal studies [12, 35], and small clinical trials [36–40]. The results showed promising reduction of postoperative scar formation. However, due to complex process of wound healing, treatment with one target remains insufficient for GFS. There have been reports regarding the clinical use of BVZ in combination with 5-Fu or MMC during GFS [41–43]. Nevertheless, the effects of combined application of anti-VEGF antibody and antimetabolite on HTFs and vascular endothelial cells are worth studying. There are problems on whether these show a better performance in the inhibition of wound scar formation after GFS, as well as their safety and underlying mechanisms.

This study initially examines the cytotoxicity of BVZ, BVZ combined with 5-Fu, and BVZ combined with MMC drugs in HTFs and HUVECs. The results revealed that low concentrations of BVZ have relatively low cellular toxicity, and the toxicity of BVZ in HTF cells is significantly increased with increasing concentrations. According to the previous reports, this cytotoxicity might be explained by nonspecific IgG-related toxicity [44]. Therefore, a relatively low concentration of BVZ is selected for detecting the cytotoxicity of combination of drugs in HTFs, while a relatively high concentration of BVZ is selected in HUVECs. The cytotoxicity of combined drug is significantly greater than that of single drug. This is different from the results of our previous research study [45]. However, previous experiments evaluated the effects of BVZ in retinal pigment epithelial (RPE) cells. Therefore, the results of this study suggests that the combined application of BVZ and antimetabolites, especially by subconjunctival injection, is considered to be safer when administering at different sites (such as the bilateral sides of the filtering bleb) or at different time points.

This experiment shows that the combination of anti-VEGF antibody BVZ and antimetabolites can inhibit scar formation of filtering bleb after GFS. This involves direct inhibition of HTF proliferation and collagen formation. HTF is regarded as an important mediator during the formation of subconjunctival scar after trabeculectomy [23]. The scratch-wound assay is used to observe the effects of combined drugs on HTF migration. Meanwhile, their effects on the expression of Col1A1 in HTF are detected. The relative cell migration rate of HTFs in BVZ + 5-Fu group is significantly lower than that of BVZ or 5-Fu groups but was not significantly different from MMC or BVZ + MMC group (i.e., BVZ + 5-Fu group and MMC or BVZ + MMC group). Similar results are observed by qPCR analysis of Col1A1 mRNA. This meant that the direct inhibitory ability of BVZ + 5-Fu on scar formation is significantly higher than that of BVZ or 5-Fu, which is comparable with that of MMC. However, the inhibitory ability of BVZ + MMC is not greater than that of MMC. The results of this in vitro experiment are consistent with the results of animal experiments [45]. On the other hand, inhibition of filtering bleb scar formation after GFS by combining BVZ and antimetabolites involves indirect inhibition. Increased angiogenesis is associated with filtration failure [46, 47]. Vascular endothelial cells are important cells involved in the process of angiogenesis during wound healing process [48]. Decreased VEGF levels in vascular endothelial cells inhibit the formation of new blood vessels at the trabeculectomy site and reduce the permeability of blood vessels, showing an indirect inhibitory effect on scar formation. The combined drugs have significant inhibitory effects on VEGF levels in HUVECs, which are significantly greater than the use of single-drug antimetabolites.

VEGF is one of the key regulators of angiogenesis, vasculogenesis, and developmental hematopoiesis. VEGF is a mitogen and acts as a survival factor for vascular endothelial cells, promoting vascular endothelial cells and monocyte movement [49, 50]. VEGF receptors mainly include VEGFR1 (Flt-1) and VEGFR2 (KDR) [51]. VEGFR2 is regarded as a major receptor of VEGF and plays a role in mitosis, angiogenesis, and permeability. VEGFR1 is a negative regulator of VEGF during the early developmental stage. VEGFR1 regulates the concentration of free ligands near cells by blocking the binding of VEGF to VEGFR2 in the form of decoy receptors during embryonic stage [50, 51]. BVZ downregulates cell mitosis and angiogenesis by binding to the transmembrane tyrosine kinase receptors VEGFR1 and VEGFR2 [52]. This study demonstrates that BVZ combined with antimetabolites significantly inhibits the expression of VEGF (R) mRNA in HTFs. The effects of combined drugs are greater than those of 5-Fu or MMC alone. BVZ + MMC has a stronger inhibitory effect on VEGFR1 mRNA expression in HTFs than BVZ + 5-Fu. For VEGFR2 mRNA expression in HTFs, the inhibitory effect of combined drugs is greater than that of BVZ, while the effects of BVZ, BVZ + MMC, and BVZ + 5-Fu are comparable in inhibiting VEGF mRNA formation in HTFs. Further research regarding the mechanisms of combined drugs is warranted.

This is an in vitro study conducted to observe the safety of BVZ combined with 5-Fu and BVZ combined with MMC and to evaluate their inhibitory effects on scar formation after GFS. Our experiments suggest that the antiscarring effect of BVZ combined with 5-Fu is more significant than that of BVZ or 5-Fu alone, which is comparable with MMC. However, the inhibitory effects on scar formation by BVZ combined with MMC are not greater than MMC. In addition, the results of our experiments suggest that combination of BVZ and antimetabolites at different sites (such as subconjunctival injection on bilateral sides of the filtering bleb) or time points are safer when considering their cytotoxicity. Our research also contributes to a more comprehensive understanding regarding the role of BVZ combined with antimetabolites during wound healing process.

## Figures and Tables

**Table 1:** Human Tenon's fibroblasts (HTFs) and human umbilical vein endothelial cells (HUVECs) were incubated with different concentrations of bevacizumab (BVZ), 5-fluorouracil (5-Fu), BVZ/5-Fu, mitomycin C (MMC), and BVZ/MMC for cytotoxicity analyses.

	Medicine and concentration (mg/ml)		Medicine and concentration (mg/ml)	
HTF	BVZ	0.025		
		0.25		
		2.5		
	5-Fu	0.05	BVZ 0.25/5-Fu	0.05
		0.5		0.5
		5		5
	MMC	0.00002	BVZ 0.25/MMC	0.00002
		0.0002		0.0002
		0.002		0.002
HUVEC	BVZ	0.025		
		0.25		
		2.5		
	5-Fu	0.05	BVZ 2.5/5-Fu	0.05
		0.5		0.5
		5		5
	MMC	0.00002	BVZ 2.5/MMC	0.00002
		0.0002		0.0002
		0.002		0.002

**Table 2:** Primers used in real-time polymerase chain reaction.

Gene name	Primer sequences
VEGF	Forward: 5ʹ-ATCGAGTACATCTTCAAGCCAT-3ʹ
	Reverse: 5ʹ-GTGAGGTTTGATCCGCATAATC-3ʹ
Flt-1	Forward: 5ʹ-CAAGATTTGCAGAACTTGTGGA-3ʹ
	Reverse: 5ʹ-CTGTCAGTATGGCATTGATTGG-3ʹ
KDR	Forward: 5ʹ-GGAGCTTAAGAATGCATCCTTG-3ʹ
	Reverse: 5ʹ-GATGCTTTCCCCAATACTTGTC-3ʹ
Col1A1	Forward: 5ʹ-AAAGATGGACTCAACGGTCTC-3ʹ
	Reverse: 5ʹ-CATCGTGAGCCTTCTCTTGAG-3ʹ
GAPDH	Forward: 5ʹ-AGACAGCCGCATCTTCTTGT-3ʹ
	Reverse: 5ʹ-CTTGCCGTGGGTAGAGTCAT-3ʹ

**Table 3:** Cell viability of human Tenon's fibroblasts (HTFs) after treatment with bevacizumab (BVZ), 5-fluorouracil (5-Fu), BVZ/5-Fu, mitomycin C (MMC), and BVZ/MMC.

Medicine and concentration (mg/ml)		Absorbance at 570 nm (ratio, compared to PBS)	Medicine and concentration (mg/ml)		Absorbance at 570 nm (ratio, compared to PBS)
BVZ	0.025	1.0596 ± 0.0307			
	0.25	0.7871 ± 0.1008			
	2.5	0.2376 ± 0.0084			
5-Fu	0.05	1.0161 ± 0.0335	BVZ0.25/5-Fu	0.05	0.6626 ± 0.1499
	0.5	0.8961 ± 0.0284		0.5	0.7962 ± 0.0365
	5	0.8417 ± 0.0258		5	0.7426 ± 0.0270
MMC	0.00002	1.0994 ± 0.0461	BVZ0.25/MMC	0.00002	0.4837 ± 0.1337
	0.0002	1.0390 ± 0.0250		0.0002	0.5255 ± 0.0942
	0.002	0.9512 ± 0.0266		0.002	0.4980 ± 0.1015

**Table 4:** Cell viability of human umbilical vein endothelial cells (HUVECs) after treatment with bevacizumab (BVZ), 5-fluorouracil (5-Fu), BVZ/5-Fu, mitomycin C (MMC), and BVZ/MMC.

Medicine and concentration (mg/ml)		Absorbance at 570 nm (ratio, compared to PBS)	Medicine and concentration (mg/ml)		Absorbance at 570 nm (ratio, compared to PBS)
BVZ	0.025	1.0652 ± 0.0792			
	0.25	1.0030 ± 0.0598			
	2.5	1.1882 ± 0.0699			
5-Fu	0.05	0.9645 ± 0.0739	BVZ2.5/5-Fu	0.05	0.7194 ± 0.0929
	0.5	0.9938 ± 0.0492		0.5	0.6249 ± 0.0701
	5	0.8967 ± 0.0172		5	0.4385 ± 0.0108
MMC	0.00002	1.1165 ± 0.0726	BVZ2.5/MMC	0.00002	0.5989 ± 0.0734
	0.0002	1.0582 ± 0.1162		0.0002	0.6698 ± 0.0960
	0.002	0.9254 ± 0.0676		0.002	0.6613 ± 0.1846

**Table 5:** Multiple comparison analysis showing the differences in the relative cell migration rate of human Tenon's fibroblasts (HTFs) in each group.

Group	BVZ + 5-Fu	5-Fu	BVZ	BVZ + MMC	MMC	VEGF	Con
	*P* value	*P* value	*P* value	*P* value	*P* value	*P* value	*P* value
BVZ + 5-Fu	—	0.001	<0.0001	0.066	0.099	<0.0001	<0.0001
5-Fu	0.001	—	0.001	0.019	<0.0001	<0.0001	<0.0001
BVZ	<0.0001	0.001	—	0.002	<0.0001	<0.0001	<0.0001
BVZ + MMC	0.066	0.019	0.002	—	0.027	<0.0001	<0.0001
MMC	0.099	<0.0001	<0.0001	0.027	—	<0.0001	<0.0001
VEGF	<0.0001	<0.0001	<0.0001	<0.0001	<0.0001	—	<0.0001
Con	<0.0001	<0.0001	<0.0001	<0.0001	<0.0001	<0.0001	—

*Note*. BVZ: bevacizumab; 5-Fu: 5-fluorouracil; MMC: mitomycin C; VEGF: vascular endothelial growth factor; Con: control.

## Data Availability

All the data supporting the findings of this study are available within the article.
